# Primary microcephaly case from the Karachay-Cherkess Republic poses an additional support for microcephaly and Seckel syndrome spectrum disorders

**DOI:** 10.1186/s12920-018-0326-1

**Published:** 2018-02-13

**Authors:** Andrey V. Marakhonov, Fedor A. Konovalov, Amin Kh. Makaov, Tatyana A. Vasilyeva, Vitaly V. Kadyshev, Varvara A. Galkina, Elena L. Dadali, Sergey I. Kutsev, Rena A. Zinchenko

**Affiliations:** 1 Research Centre for Medical Genetics, Moscow, Russia; 2 Moscow Institute of Physics and Technology, Dolgoprudny, Russia; 3 Genomed Ltd, Moscow, Russia; 4 Khabez central district hospital, Khabez, Russia; 5 Pirogov Russian National Research Medical University, Moscow, Russia; 6 Moscow State University of Medicine and Dentistry, Moscow, Russia; 7 Laboratory of Genetic Epidemiology, Research Centre for Medical Genetics, Moskvorechie St., 1, Moscow, Russian Federation 115478

**Keywords:** ASPM, Clinical continuum, Clinical heterogeneity, Allelic disorders, Seckel syndrome

## Abstract

**Background:**

Primary microcephaly represents an example of clinically and genetically heterogeneous condition. Here we describe a case of primary microcephaly from the Karachay-Cherkess Republic, which was initially diagnosed with Seckel syndrome.

**Case presentation:**

Clinical exome sequencing of the proband revealed a novel homozygous single nucleotide deletion in *ASPM* gene, c.1386delC, resulting in preterm termination codon. Population screening reveals allele frequency to be less than 0.005. Mutations in this gene were not previously associated with Seckel syndrome.

**Conclusions:**

Our case represents an additional support for the clinical continuum between Seckel Syndrome and primary microcephaly.

## Background

Primary, or congenital, microcephaly (MCPH) is characterized by a decrease in the head circumference more than four standard deviations (SD) below age and sex-specific means [1]. Often, microcephaly is accompanied by a psychomotor retardation. Primary microcephaly could be caused by either hereditary or environmental factors, including maternal exposure to toxoplasma or Zika virus [2], to alcohol or excessive amounts of the phenylalanine [3, 4]. The presence of facial dysmorphism points at the need for differentiating this condition from the Seckel syndrome as well as from lissencephaly and Rubenstein-Taybi and Norman-Roberts syndromes. Hereditary primary microcephaly is a genetically heterogeneous group of conditions inherited mainly in autosomal recessive mode, though several dominant forms have been described [5]. Although MCHP and Seckel syndrome were previously distinguished by height (maximum height in Seckel syndrome was equivalent to the minimum height in MCPH), stature is no longer a discriminating feature, leading to the conclusion that these phenotypes constitute a spectrum rather than distinct entities [6]. The Seckel syndrome is characterized by more severe intellectual disability as well as more often the presence of characteristic facial features. To date, 17 different genes associated with autosomal recessive MCPH are identified. Nine genes are associated with Seckel syndrome, of them 2 (*CENPJ* and *CEP152*) could cause both MCPH and Seckel syndrome.

In consanguineous populations, the prevalence of primary microcephaly was estimated to be 1 in 10,000—6.8 per 10,000 [7]. Homozygous and compound heterozygous mutations in *ASPM* gene (MCPH5; OMIM #605481) account for up to 40% of primary MCPH cases in both consanguineous and non-consanguineous families [8]. ASPM (Abnormal Spindle Microtubule Assembly) protein is a part of a mother centriole complex; it regulates centriole biogenesis during neurogenesis, apical complex, and cell fate [9].

Here we present a case of primary microcephaly with family recurrence. This case was found in Khabezsky district of the Karachay-Cherkess Republic, Russia, inhabited by approximately 30,000 dwellers of predominantly Circassian origin (95.2%).

The Circassians belong to the Northwest Caucasian ethnic group [10] speaking the mutually intelligible continuum of Circassian language with two literary standards, Adyghe (West Circassian) and Kabardian or Kabardino-Cherkess (East Circassian). In its narrowest sense, the term “Circassian” is restricted to twelve Adyghe tribes [11]. Importantly, documented calamities of the 19th and 20th centuries, including the Caucasian War of 1817–1864, resulted in the forcible eviction of a large part of the Circassians into the Ottoman Empire. Further administrative transformations carried out by the tsarist government and then by the Soviet authorities led to the formation of four territorially isolated groups of the Circassian people, with separate ethnographic designations: Kabardian (Circassians of the Kabardino-Balkar Republic), Cherkess (Circassians of the Karachay-Cherkess Republic), Adyghe (Circassians of the Kuban including the Republic of Adygea and Krasnodar Krai), and Shapsug (the indigenous historical inhabitants of Shapsugia) [12]. These four Circassian populations, including northwestern Adyghe people, do not differ in common mtDNA haplogroup frequencies [13]. The Y-chromosomal markers data suggested a direct origin of Caucasus male lineages from the Near East, followed by high levels of isolation, differentiation and genetic drift in situ [14].

## Case presentation

**Fig. 1:**
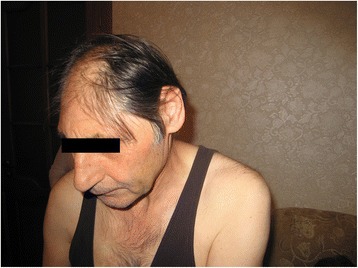
Proband’s phenotype

**Fig. 2:**
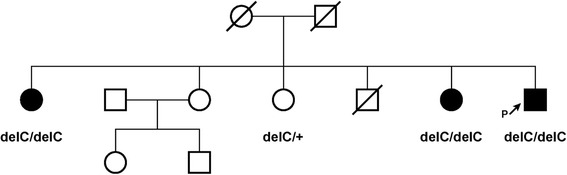
Pedigree of the family

Here we describe a Circassian family with three affected siblings: a proband (eхamined at the age of 66 years old), and his two sisters, examined at the age of 58 and 56 years old, all were ascertained with the primary incoming diagnosis of Seckel syndrome. The family also included three healthy siblings, two sisters and a brother. The patients were examined during a field expedition to the Karachay-Cherkess Republic with the help of local Ministry of Health Care. Detailed clinical examination detected following phenotypic features: mental retardation, marked decrease in the circumference of the head (proband and one siblings – 46 cm, another sib – 44 cm), pronounced predominance of the facial part of the skull over the cerebral, large protruding low-set ears, narrow beveled forehead, low hair growth on the forehead, high roof of the mouth, microgenia, muscular hypertonus, contractures in the elbow joints without pathological reflexes (Fig. 1). Epileptic seizures were not observed. All affected family members also demonstrated short stature (142–144 cm), kyphoscoliosis (1–2 degree), and a serious deficiency of the cognitive component of behavior with the preservation of the response to simple commands (eating, taking hygienic procedures). They have no reading, writing, and arithmetic skills, and demonstrated monosyllabic speech resembling that of a 3–4 years old children. Archival medical records have indicated that all these children were born with low weight (below 3000 g), while their skull circumferences were at the lower limit of the norm until 6–7 months of life, with progressive declines in its percentile observed subsequently. Developmental milestones were, at first, correspondent to the age. The delay, then the stop in the growth of the cerebral cranium was observed at by 5 years, with the lag at − 4 SD. The height of healthy father and brother were at 190 cm and above. One healthy sister has height of 176 cm, while other – of 171 cm. One of the healthy sisters gave birth to healthy children (Fig. 2).

**Fig. 3:**
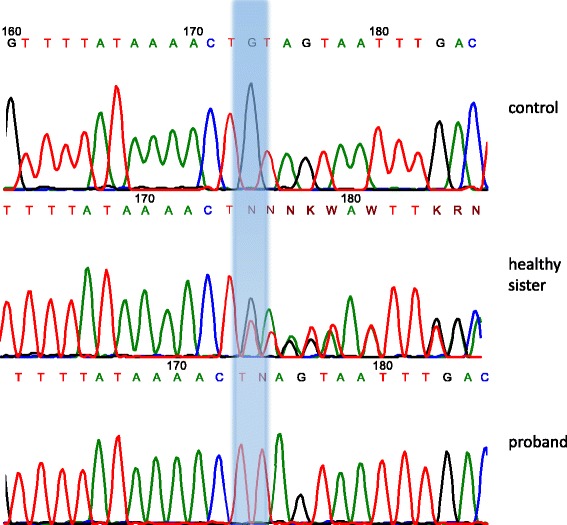
Results of Sanger sequencing

Due to the known genetic heterogeneity of Seckel syndrome, DNA diagnosis in the proband was carried out by targeted high-throughput sequencing (HTS) of clinically relevant genes (clinical exome sequencing, CES). CES was performed on Illumina NextSeq 500 instrument in 2 × 151 bp paired-end mode. A total of 13.7 million reads were obtained, corresponding to 99.9× on-target average sequencing depth based on TruSight One Sequencing Panel target region list. The raw sequencing data have been processed with a custom pipeline based on popular open-source bioinformatics tools BWA, Samtools, Vcftools, as well as in-house Perl scripts, using hg19 assembly as a reference sequence. In total 49,772 nucleotide variants were found. Variant annotations were added by SnpEff/SnpSift software using public databases (dbSNP, ExAC, ClinVar, dbNSFP). After filtering the variants by functional consequence and population frequencies, no suitable candidates were found in a proband among the genes known to date that are responsible for Seckel syndrome. After ranking the variants by their functional consequences and population frequencies, only one suitable candidate gene, *ASPM*, was identified in a proband as previously not described homozygous variant hg19::chr1:197111995TG>T. This variant leads to mutation NM_018136.4(*ASPM*_v001):c.1386delC in the exon 3 of the *ASPM* gene, leading to the formation of the premature stop codon p.Tyr462*. Sanger sequencing confirmed that two affected sisters bear the same mutation in the homozygous state while healthy siblings were heterozygous for the mutation (Fig. 3).

Importantly, homozygous and compound heterozygous loss-of-function mutations in the *ASPM* gene were previously described in patients with autosomal recessive primary MCPH type 5 (OMIM #608716). *ASPM*:p.Tyr462* mutation has not been previously found in the publicly available control cohorts (genome Aggregation Database) as well as in 202 population-matched control chromosomes (screened by PCR-RFLP). Therefore, we conclude that, according to the ACMG criteria, on the strength of cumulative evidence, this mutation should be regarded as pathogenic [15]. As the mutation causes the formation of the premature stop codon – p.Tyr462* – the mRNA should be a target for nonsense-mediated mRNA decay (NMD) leading to the null-allele [16].

As this mutation occurred in the homozygous state in the proband, estimations of run-of-homozygosity (ROH) region length were performed around the mutation according to the states of alternate alleles of frequent SNPs covered by clinical exome sequencing data. We found that in this Circassian family, ROH region spreads at least from rs79351096 to rs4950927, with the minimal length 6.2 Mb. In fact, the length of ROH region could be even greater as clinical exome data used for its estimation cover only coding sequences of genes related to hereditary diseases.

## Discussion

Here we present a description of a Circassian family with three out of six siblings displaying primary microcephaly, short stature, mental retardation, and bird-like face. Clinical exome sequencing revealed a novel homozygous single nucleotide deletion c.1386delC in *ASPM* gene, which leads to preterm stop-codon and truncating of protein. According to The American College of Medical Genetics and Genomics (ACMG) criteria, this single nucleotide variant is classified as pathogenic with a strong evidence (PM2, PVS1, PS3, PP1-S) [15]. The same ethnic background of the parents of the index patient could explain the homozygous state of the identified mutation. However, population screening for the mutation in 202 normal chromosomes reveals no carriership, indicating that the frequency of this mutation is less than 0.005. Analysis of the genetic structure of the Circassian population shows that in the rural district of the family’s residence the level of random Wright inbreeding (*F*_ST_) was at 0.00890, while the value of local inbreeding estimated through the isolation model by the Malecot’s distance was at 0.00933, i. е. almost 1% [17, 18]. In addition, it is known that the marriages with a positive ethnic assortativeness are preferred in this population. Although the pedigree does not show the consanguinity, taking into account the genetic structure of the population, we should assume the presence of consanguinity [19]. Analysis of runs-of-homozygosity on CES data also supports the idea of the inbred origin of the proband. The length of ROH region encompassed the revealed homozygous frame-shifting deletion appears to be at least 6.2 Mb, which is much greater than an average for outbred populations [20], thus, pointing to the possible endogamous ancestry of the family.

To date, more than 400 different nucleotide variants in ASPM gene are registered in ClinVar [21], and only 155 of them reported to be pathogenic or likely pathogenic. A majority of them being loss-of-function and should lead to NMD. All reported mutations of *ASPM* are associated with autosomal recessive primary MCPH type 5. To date, 17 genes are described to be associated with primary autosomal recessive MCPH. The vast majority of them participate in mitotic spindle assembly (*ASPM*, *WDR62*, *CDK5RAP2*, *KNL1*, *CENPJ*, *STIL*, *CEP135*, *CEP152*, *CENPE*, *SASS6*, *CIT*, and *ANKLE2*), while others are associated with chromosome condensation and maintenance (*MCPH1*, *ZNF335*, *PHC1*), cell cycle control (*CDK6*), and blood-brain barrier maintenance (*MFSD2A*). Mutations in two of them, *CENPJ* and *CEP152*, could also cause an allelic condition known as autosomal recessive Seckel syndrome [22, 23], which is characterized by proportionate growth and mental retardation, microcephaly, and characteristic bird-like face. Other forms of Seckel syndrome are caused by mutations in genes associated with cell growth (*TRAIP*), genomic integrity and repair (*ATR*, *NSMCE2*, *DNA2*, and *RBBP8*), centrosome function (*NIN*, *CEP63*) [6]. Clinical diagnosis of these conditions is also complicated by the need to differentiate them from primordial dwarfism which sometimes could lead to similar phenotypes [24], but may be distinguished from Seckel syndrome by radiological assessment. Meier-Gorlin syndrome could also manifest with microcephaly and intrauterine and postnatal growth retardation [25]. This clinical spectrum of overlapping phenotypes makes differential diagnosis challenging.

## Conclusions

The proband presented here was initially diagnosed with Seckel syndrome because of primary microcephaly, severe mental delay, and characteristic facial features. This phenotype is not common in described primary microcephaly cases as intellectual disability is usually more severe in Seckel syndrome as well as characteristic facial features, which could correspond to the relative sparing of the midfacial structures compared to the rest of the head. High-throughput sequencing of clinically relevant genes in proband identified no candidate nucleotide variants in any genes associated with Seckel syndrome to date. The only mutation identified in this family was a frame-shifting single nucleotide deletion affecting *ASPM* gene. To our knowledge, no *ASPM* mutations have been associated with Seckel-like phenotypes to date. Therefore, our observation broadens the phenotypic heterogeneity of MCPH and supports the view on MCPH and Seckel syndrome as a clinical continuum.
